# Biomaterials in Cancer Therapy: Investigating the Interaction between Kaempferol and Zinc Ions through Computational, Spectroscopic and Biological Analyses

**DOI:** 10.3390/ma17112526

**Published:** 2024-05-24

**Authors:** Aleksandra Golonko, Adam Jan Olichwier, Adam Paszko, Renata Świsłocka, Łukasz Szczerbiński, Włodzimierz Lewandowski

**Affiliations:** 1 Clinical Research Centre, Medical University of Bialystok, 15-276 Bialystok, Poland; 2 Prof. Wacław Dąbrowski Institute of Agricultural and Food Biotechnology—State Research Institute, 02-532 Warsaw, Poland; r.swislocka@pb.edu.pl; 3 Department of Chemistry, Biology and Biotechnology, Bialystok University of Technology, 15-351 Bialystok, Poland; 4 Department of Endocrinology, Diabetology and Internal Medicine, Medical University of Bialystok, 15-276 Bialystok, Poland

**Keywords:** coordination chemistry, computational drug design, cancer therapy, zinc complex bioactivity, molecular spectroscopy

## Abstract

A complex of the natural flavonoid kaempferol with zinc (Kam-Zn) was synthesized, and its physicochemical properties were investigated using spectroscopic methods such as Fourier transform infrared spectroscopy (FT-IR), ultraviolet–visible (UV-Vis) spectroscopy and theoretical chemistry. Biological studies were conducted to evaluate the cytotoxic and antiproliferative effects of these complexes on MCF-7 breast cancer cells. Treatment with Kam 100 µM (84.86 ± 7.79%; 64.37 ± 8.24%) and Kam-Zn 100 µM (91.87 ± 3.80%; 87.04 ± 13.0%) showed no significant difference in proliferation between 16 h and 32 h, with the gap width remaining stable. Both Kam-Zn 100 μM and 200 μM demonstrated effective antiproliferative and cytotoxic activity, significantly decreasing cell viability and causing cell death and morphology changes. Antioxidant assays revealed that Kam (IC50 = 5.63 ± 0.06) exhibited higher antioxidant potential compared to Kam-Zn (IC50 = 6.80 ± 0.075), suggesting that zinc coordination impacts the flavonoid’s radical scavenging activity by the coordination of metal ion to hydroxyl groups. Computational studies revealed significant modifications in the electronic structure and properties of Kam upon forming 1:1 complexes with Zn^2+^ ions. Spectroscopy analyses confirmed structural changes, highlighting shifts in absorption peaks and alterations in functional group vibrations indicative of metal–ligand interactions. FT-IR and UV-Vis spectra analysis suggested that Zn coordinates with the 3-OH and 4C=O groups of ligand. These findings suggest that the Kam-Zn complex exhibits interesting antiproliferative, cytotoxic and modified antioxidant effects on MCF-7 cells, providing valuable insights into their structural and anticancer properties.

## 1. Introduction

Kaempferol (Kam) is a natural flavonoid, which belongs to a group of tetrahydroxyflavones, as it has four hydroxy groups positioned at 3, 5, 7 and 4′ [1]. Kam is found in products of plant origin, mainly leafy vegetables such as spinach in the amount of 55 mg/100 g of raw material and broccoli at 7.2 mg/100 g but also in capers, on average 104.29 mg/100 g [2]. Many studies have shown that Kam has various positive features, including antidiabetic, antioxidant, anti-inflammatory, anticancer, neuroprotective and cardioprotective activities. Kam is a flavonoid widely present in many plants, including medicinal raw materials used in health-supporting therapies. Its pleiotropic effect, which influences various biological pathways and processes in the human body, makes it an interesting subject for scientific research. The structural analysis of kaempferol and its ability to form complex compounds with micro and macro elements naturally occurring in the human body could help elucidate the mechanisms of action of this flavonoid at the molecular level, which is crucial for fully leveraging its therapeutic potential. The therapeutic use of Kam is limited by its low solubility, which is why many studies focus on modifying the structure of this ligand in order to improve bioavailability and biological activity, e.g., by synthesizing complex compounds with metal ions [3,4,5] or nanoparticles [4,6,7]. The attention of recent studies focuses on its potential use in cancer therapy, as increased intake was found to decrease likelihood of many types of cancer like stomach, liver, ovary and skin cancer [8]. Its anticancer activity in hormone-dependent cancer, observed specifically in estrogen-dependent breast cancer cells, seems to be particularly interesting, as Kam can regulate the expression and function of estrogen receptor alpha (Erα) [9]. In addition, the observed dualistic nature of Kam estrogenic (in low concentrations) and anti-estrogenic (in higher concentrations) effects may explain its potential benefits in the prevention of diseases associated with estrogen imbalance [10]. Additionally, Kam has been found to inhibit estrogen receptor alpha expression and function in MCF-7 cells, leading to a dose-dependent decrease in cell number and a concomitant decrease in ER-alpha protein levels, further supporting its antiproliferative effects [11].

Another mechanism that determines the cytotoxic nature of this compound against breast cancer cells may be the inhibition of glucose uptake, which supports a high rate of glycolysis and uncontrolled proliferation [12]. Among the mechanisms identified for the pro-apoptotic properties of Kam so far are the induction of the mitochondrial caspase-9 pathway, activation of PARP, blockade of PKC/MAPK/AP-1 signaling and inhibition of MMP expression and activity [13].

Moreover, its antioxidant action is also studied in reversing the cell phenotype of malignant cancers due to its ability to inhibit reactive oxygen species (ROS) [14]. The coordination of a metal ion by a flavonoid causes a change in the electron charge of the molecule, which affects the acceptor–donor properties and thus the antiradical activity and the ability to bind to biological macromolecules [15,16,17].

Considering the range of pleiotropic properties of Kam, many studies have utilized this ligand in the synthesis of complex compounds to enhance or impart new biological properties, including cytotoxicity. Kam-Zn complexes, particularly those like [Zn(Kam)(phen)]Cl·H_2_O and [Zn(Kam)(bpy)]Cl·H_2_O, demonstrate notable anticancer potential. In studies involving the MCF-7 breast cancer cell line, these complexes have shown a significantly higher cytotoxic effect compared to that of ligand alone [18]. Their mechanisms include DNA intercalation and apoptosis induction, which are critical for their enhanced therapeutic efficacy [19]. The coordination of flavonoids with transition metals such as copper and iron has resulted in complexes with enhanced stability and biological activity [20]. Additionally, the apoptotic effect of a Zn(II) complex with an N-donor heterocyclic ligand on MCF-7 cells was significant, with notable induction of apoptotic cell death and S-phase arrest, highlighting the complex’s therapeutic potential [19], but underlying molecular mechanisms of Zn(II) complex-based apoptotic cell death need to be explored. Its complexes, particularly those with nitrogen donor ligands, have gained attention due to their potential antitumor activities, generally exerting lower toxicity and offering fewer side effects compared to other metal-based drugs [21]. Due to the fact that chemotherapy is very likely to impair the health of cancer patients, the attention of many researchers is focused on the utilization of substances, which might alleviate the side effects of cancer therapy with chemo pharmaceuticals [22]. Despite some controversies, which were very probably the result of a lack of clinical trials, antioxidants are promising compounds that may play a protective role in alleviating the side effects of chemotherapy [23]. Other metals like platinum, ruthenium, gold and palladium have been utilized in designing anticancer drugs. For instance, platinum(IV) complexes with modifications, such as incorporating ferrocene or monoaminophosphate moieties, have shown enhanced solubility, stability and antiproliferative activity against lung cancer cells, with reduced toxicity to normal cells. Heteronuclear Pt(IV)-Ru(II) complexes, in particular, have exhibited cytotoxic and anti-metastasis properties, leveraging the advantages of two metal centers [24].

Zinc belongs to the IIB group of metals, and its essential role in biologic processes in humans was first described about 60 years ago [25]. Zn participates in hundreds of physiological reactions because the action of more than 300 enzymes and proteins requires the presence of Zn for catalytic activity, e.g., superoxide dismutase (SOD) enzymatic antioxidant or caspase, a Zn-dependent proteolytic enzyme with an important role in apoptosis [26]. Zn is particularly associated with the proliferation and differentiation of cells and also significantly participates in metabolic reactions of carbohydrates and lipids [27]. Zn deficiency might be related to Alzheimer disease, Parkinson disease, diabetes, impaired wound healing, cancer or stroke [28]. Flavonoids were found to be able to regulate the uptake, transport and homeostasis of Zn ions [29].

The chemical structure of flavonoids contains phenolic and carbonyl groups, which function as possible sites for the chelation of Zn and other metals [30], but the exact mechanism for the modulation of antioxidant properties of flavonoids is yet to be discovered. The topic of biological activity of flavonoids and metal complexes is widely discussed in the context of their therapeutic use, as well as in combination therapy with conventional cytostatic pharmaceuticals [31,32].

## 2. Materials and Methods

### 2.1. Synthesis and Chemicals

Kam (Sigma Aldrich, CAS: 520-18-3, Burlington, MA, USA) was dissolved in 96% methanol to prepare a stock solution. Separately, zinc chloride (CAS: 231-592-0, anhydrous) was dissolved in methanol to obtain a methanolic solution. The two solutions were combined in a 1:2 molar ratio (Kam/zinc chloride) to form the Kam-Zn complex. The resulting solution was allowed to precipitate, followed by washing with methanol on filter paper to remove any residual chloride ions. The absence of chloride ions in the filtrate was confirmed by the silver nitrate test. For the antioxidant assay, the Kam-Zn complex solution was rapidly dissolved in dimethyl sulfoxide (DMSO) to obtain a 0.04 M stock solution. Working solutions were prepared by diluting the stock solution directly in the appropriate culture medium. The Kam-Zn complex obtained after precipitation and washing was used for the FT-IR spectroscopy assay. The complex was characterized by recording its FT-IR spectrum and comparing it to the spectrum of Kam alone, revealing significant differences in the spectra and providing insights into the structural changes and coordination sites of the zinc ion. The DPPH solution was prepared by weighing 24 mg of the solid reagent 2,2-diphenyl-1-picrylhydrazyl into 100 mL of methanol. This initial solution was then diluted fivefold in methanol. For the assay, 15 µL of the test solutions in 0.04 µM were applied to a 96-well plate with a transparent bottom, and 285 µL of the working DPPH (0.12 mmol/L) reagent was added.

### 2.2. Computational Studies

Calculation was carried out using the B3LYP/6-311++G(d,p) method. The electron charge distribution was calculated for the optimal structures by the NBO (natural bond orbital) method. The energy of the HOMO and LUMO orbitals and the reactivity descriptors were calculated using the B3LYP/6-311++G(d,p) method. Calculations were made using the B3LYP/6-311++G(d,p) density functional method. All theoretical calculations were performed using the Gaussian 09 program package (Gaussian, Inc., Wallingford, CT, USA, 2016). Additionally, the WebMO application was used to visualize selected electrophilic and nucleophilic surfaces [33]. The following expressions were used to describe changes in global reactivity descriptors [34]: electronegativity $\chi =-\frac{1}{2}(I+A)$; global hardness $\eta =-\frac{1}{2}(I-A)$; global softness $S=\frac{1}{\eta }$; chemical potential $\mu -\frac{1}{2}\left(I+A\right)$; and electrophilicity $\omega =-\frac{\chi^{2}}{2\eta }$. The formulas use ionization energy (*I*) and electron affinity (*A*), where *I* = HOMO energy [eV] and *A* = LUMO energy [eV].

### 2.3. Spectroscopy Studies

#### 2.3.1. UV-Vis Spectroscopy

UV-Vis spectroscopy was used to study the interaction between Zn^2+^ ions and Kam. This interaction was assessed by measuring the absorption spectrum between 190 and 500 nm in a methanolic solution of the ligand, prepared at a concentration of 1.0 × 10^−4^ M in 100% methanol. A Zn^2+^ ion solution was added to the kaempferol solution in a 1:1 ratio, and after 45 min, a second Zn^2+^ ion solution was added to achieve a 1:2 ratio.

#### 2.3.2. FT-IR Spectroscopy

FT-IR spectra were recorded using a BRUKER ALPHA (Billerica, MA, USA) spectrometer equipped with a universal transmission adapter. Samples were prepared in a KBr matrix at a 200:1 ratio. The spectra were measured in the wavenumber range of 4000–400 cm^−1^ with a resolution of 4 cm^−1^. Theoretical calculations were performed using the Gaussian 09 program package, applying a vibrational frequency scaling factor (IR factors) of 0.98 for the calculated IR spectra.

#### 2.3.3. Job’s Method of Continuous Variations

Job’s method of continuous variations is a commonly used technique in coordination chemistry to determine the stoichiometry of metal–ligand complexes. In this method, solutions of Zn^2+^ and a ligand are mixed in different proportions while keeping the total volume of the mixture constant at 2 mL. The XL value is calculated as the ratio of the concentrations of the metal ion and the ligand (CM/CL + CM). In this experiment, the absorption was recorded at a wavelength of 421 nm, which was determined experimentally by measuring the increase in absorbance until it reaches its maximum. The XL value corresponding to the maximum absorbance is taken as the stoichiometric ratio of the metal–ligand complex.

### 2.4. Antioxidant Properties

Kam and Kam-Zn(II) solutions were prepared in a dilution series of 0.005–40 µM by dissolving the stock solution in methanol. Solid DPPH reagent (1,1-diphenyl-2-picrylhydrazyl) (CAS: 1898-66-4) purchased from Sigma Aldrich) was dissolved in methanol to a target concentration of 4.8 mg/dm^3^ and finally kept in the dark for 2 h. Each 30 µL of sample solution was added to DPPH solution and mixed quickly. All probes were kept at room temperature, away from the light for 30 min. The absorbance of the solution at 517 nm was recorded at microplate reader. A mixed solution of DPPH and methanol instead of sample solution was used as the control. The scavenging activity was obtained from the following equation:

$$Scavenging\;Activity\;\left(\%\right)=\frac{A_{control\;}-A_{sample}}{A_{control}}\cdot 100\%,$$

where *A* is the absorbance of the sample. The IC50 value was calculated from the regression curve equation of the linear relationship between the concentration and the degree of the free-radical scavenging part.

### 2.5. Cell Line

MCF-7 human breast cancer cells (ATCC-HTB-22 MCF7; Breast Adenocarcinoma, Gaithersburg, MD, USA) were cultured in Dulbecco’s Modified Eagle’s Medium (DMEM) supplemented with 10% fetal bovine serum (FBSF9665, Fetal Bovine Serum (heat inactivated, non-USA origin, sterile-filtered, Sigma-Aldrich) and 1% penicillin–streptomycin. Cells were maintained at 37 °C in a humidified atmosphere containing 5% CO_2_.

### 2.6. Cytotoxicity Assay In Vitro

3-(4,5-Dimethylthiazol-2-yl)-2,5-diphenyltetrazolium bromide (MTT) is cell-permeable reagent that measures the metabolic activity of living cells. Only active mitochondria in living cells will lead to the cleavage of the tetrazolyl ring to formazan with the participation of oxidoreductase enzymes—in particular NADH (nicotinamide adenine dinucleotide). Formed formazan crystals were dissolved by adding DMSO and measuring the absorption at 590 nm. Control cells were exposed to 1% DMSO. Experiments were run in triplicate and repeated 3 times.

### 2.7. Antiproliferative Assay In Vitro

MCF-7 breast cancer cells were seeded in 24-well polystyrene plates from Nunc. The wound was created using a 1 mL pipette tip. Cells were washed with warm PBS and treated with DMEM supplemented with 10% FBS, 1% penicillin–streptomycin and the tested compounds. DOX was added at a concentration of 0.25 μM, Kam at 50 and 100 μM and Kam-Zn complex at 50 and 100 μM. The control group was treated with DMEM containing DMSO at a concentration of <1%, similar to the cytotoxicity tests performed using the MTT assay. Cell proliferation was assessed at 16 and 32 h using a wound-healing assay. Gap widths were measured three times for each sample at designated locations on the plate using a Leica microscope equipped with LAS X (Life Science Microscope Software, LAS X 3.0, https://www.leica-microsystems.com, accessed on 20 May 2024). The results were expressed as percentages of the initial gap width, along with means and standard deviations.

## 3. Results

### 3.1. Structure Analysis

**Figure 1:**
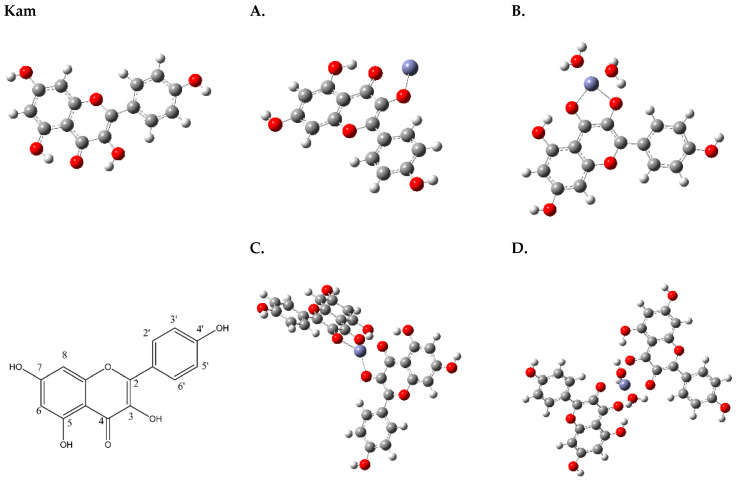
Optimized structure of Kam and general structure with atom numbering and Kam-Zn(II) metal complexes (**A**–**D**) (Obtained from Gaussian 09). White—oxygen, red—hydrogen, grey—carbon atoms, purple—zinc.

According to computational chemistry data obtained by Yadaw et al., it was determined that Kam may exist in at least several conformers, differing in the orientation of the 7-OH and 4′-OH hydroxyl groups [35]. The hydrogen atoms belonging to the 5-OH and 3-OH hydroxyl groups are oriented in such a way as to form intramolecular hydrogen bonds with =O (Figure 1).

The coordination of Kam with Zn^2+^ begins with the deprotonation of the 3-OH group on the ligand, which transforms it into a negatively charged oxygen donor capable of coordinating with Zn^2+^ (A,B). Concurrently, the oxygen atom of the 4-carbonyl group on the same ligand molecule also binds to Zn^2+^, aiding in the formation of a bidentate chelate. This results in a stable complex with Zn^2+^, potentially arranging in a tetrahedral geometry with the involvement of either another Kam molecule (C) or additional water molecules (D), where hydrogen bonding interactions can lead to more stable and specific geometric arrangements.

### 3.2. Mulliken Charges

Upon examining the Mulliken charges of the oxygen, carbon and hydrogen atoms in the uncoordinated Kam molecule and its Zn^2+^ complexes (structures A and B), we can observe significant changes in the electronic charges (Table 1) these changes in Mulliken charges are indicative of a redistribution of electron density within the molecule upon coordination with the metal ion. This redistribution of electron density can influence the reactivity of the molecules by changing donor–acceptor properties.

Based on the results obtained from Job’s method (UV-Vis), the most probable structures for the zinc complexes of Kam are structures A (Zn(Kam)) and B (Zn(Kam)(H_2_O)_2_), which exhibit a 1:1 metal-to-ligand ratio (Figure 1).

In particular, the oxygen atoms in structures A and B generally become less electron rich compared to those in the uncoordinated Kam molecule, reflecting the formation of metal–ligand bonds. The altered electron distribution around the carbon atoms upon coordination with Zn^2+^ can also impact their electron-donating or electron-accepting properties. The Mulliken charges on hydrogen atoms show smaller variations between the uncoordinated Kam molecule and the coordinated structures A and B.

In the uncoordinated Kam molecule, the oxygen atoms O1, O3, O4, O5, O7 and O4′ have negative Mulliken charges, with values ranging from −0.324 to −0.742 (atom numbering, Figure 1, Kam). Upon coordination with zinc in structure A, the Mulliken charges on these oxygen atoms generally become less negative, ranging from −0.456 to −0.715. A similar trend is observed for structure B, with Mulliken charges ranging from −0.459 to -0.607. These changes in Mulliken charges indicate that the oxygen atoms become less electron rich upon Zn coordination, which is consistent with the formation of metal–ligand bonds.

In the uncoordinated Kam molecule, the Mulliken charges on carbon atoms C2, C3, C4, C5, C6, C7, C8, C9 and C10 range from −0.633 to 0.968. In structure A, the charges on these carbon atoms generally become less positive or less negative, with values ranging from −0.568 to 0.286. Similarly, for structure B, the Mulliken charges on carbon atoms range from −0.556 to 0.251. These changes suggest that the electron distribution around the carbon atoms is altered upon coordination with zinc, potentially affecting their electron-donating or electron-accepting properties.

For the Kam molecule, the Mulliken charges on hydrogen atoms (H3, H5, H6, H7, H8, H2′, H3′, H4′, H5′ and H6′) vary between 0.106 and 0.445. In structure A, the charges on these hydrogen atoms, except H3, which is not present, range from 0.231 to 0.421. For structure B, the charges on hydrogen atoms range from 0.217 to 0.424. In both coordinated structures, the Mulliken charges on hydrogen atoms generally show small variations compared to the uncoordinated Kam molecule.

The changes in Mulliken charges and the resulting redistribution of electron density in the metal complexes compared to the ligand can lead to decreased reactivity. This decrease in reactivity can be attributed to several factors. The coordination of the ligand with the metal ion leads to the formation of metal–ligand bonds, which alters the electron distribution in the complex and stabilizes the overall structure, thereby reducing the reactivity of the coordinated ligand. The redistribution of electron density upon coordination with zinc can affect the electron-donating or electron-accepting properties of the atoms involved, making them less prone to participate in chemical reactions. The coordination of the metal ion can create steric hindrance around the ligand, which can limit the accessibility of other molecules or reagents to the reactive sites, leading to decreased reactivity.

### 3.3. Theoretical Descriptors of Structure and Physicochemical Properties

Based on the provided data, we can analyze the changes in reactivity and electronic properties between the uncoordinated Kam molecule and the structures A (Zn(Kam)) and B (Zn(Kam)(H_2_O)_2_) metal complexes (Figure 1).

The HOMO (highest occupied molecular orbital) and LUMO (lowest unoccupied molecular orbital) energy levels are associated with the molecule’s reactivity. A decrease in the HOMO–LUMO energy gap indicates increased reactivity. In this case, the energy gap for Kam is 3.711 eV, while for structure A, it is 2.57 eV, and for structure B, it is 4.65 eV. This suggests that structure A exhibits increased reactivity compared to Kam, whereas structure B has decreased reactivity.

Ionization potential represents the energy required to remove an electron from a molecule, while electron affinity measures the energy change when an electron is added to the molecule. Structure A has a lower IP (5.18 eV) compared to Kam (5.935 eV), suggesting that it is easier to remove an electron from structure A. The electron affinity for structure A (2.60 eV) is higher than that of Kam (2.224 eV), indicating that structure A has a higher tendency to accept an electron. For structure B, the IP (5.64 eV) and EA (0.99 eV) values suggest a lower reactivity compared to Kam.

Electronegativity (χ) is a measure of an atom’s ability to attract electrons. The electronegativity values for structures A (3.89 eV) and B (3.31 eV) are lower than that of Kam (4.079 eV), indicating a decreased electron-attracting ability in the metal complexes. The hardness (η) and softness (σ) values provide information about the stability and reactivity of molecules. Structure A exhibits lower hardness (1.29 eV) and higher softness (0.39 eV) compared to Kam (hardness: 1.855 eV; softness: 0.269 eV), suggesting increased reactivity. In contrast, structure B has higher hardness (2.33 eV) and lower softness (0.22 eV), indicating decreased reactivity. The electrophilicity index (ω) measures the propensity of a molecule to accept electrons. Structure A has a higher electrophilicity index (5.88 eV) than Kam (4.485 eV), while structure B has a lower electrophilicity index (2.36 eV).

**Figure 2:**
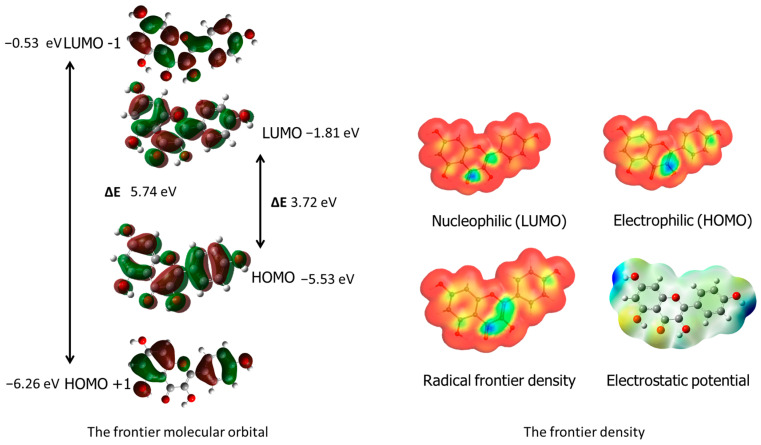
Visualization of key determinants of Kam’s reactivity. Including HOMO, LUMO, radical frontier density and electrostatic potential (obtained using the WebMO application) and HOMO, LUMO frontier orbital visualization (obtained from Gaussian 09, B3LYP/6-311++G(d,p) calculations).

The WebMO application was used to visualize radical frontier density, electrostatic potential and nucleophilic and electrophilic areas in the ligand [33]. Additionally, frontier orbital visualizations were obtained from Gaussian 09 software calculations. These results are presented in the corresponding Figure 2, providing valuable insights into the molecule’s reactivity and behavior in chemical and biological systems. The blue areas around carbon C3 indicate the protonation site, while the maximum values of the LUMO nucleophilic density around the carbonyl group carbon C4 and carbon C2 suggest the nucleophilic attack site. The molecular electrostatic potential (MEP) mapped on the electron density isosurface is also shown in Figure 2. The red-shaded areas indicate the minimum electrostatic potential regions with negative potential, while the blue color indicates the maximum electrostatic potential as the site for nucleophilic attacks.

The energy of the structures decreases as the complexity of the molecule structure increases, meaning the systems become more stable. Kam has the highest energy (−1029.04 Hartree), followed by structure A (−1093.84 Hartree) and structure B (−1246.71 Hartree). This indicates that coordination with the metal ion and water molecules stabilizes the overall energy of the system.

B3LYP energy represents the total electronic energy calculated using the B3LYP method, a hybrid functional that combines Hartree–Fock and density functional theory (DFT) approaches. The B3LYP energy includes contributions from electronic, nuclear and nuclear-electron interactions. Lower (more negative) energy values indicate greater stability of a molecule. Enthalpy decreases and entropy becomes more negative from Kam to structure A and B, indicating a more stable and ordered system in the coordinated complexes compared to the uncoordinated ligand (Kam).

The rotation constants A, B and C (Table 2) are parameters used to describe the rotational behavior of a molecule in space. These constants can be obtained from theoretical calculations in computational chemistry. A, B and C are measured in units of frequency, typically in gigahertz (GHz). A corresponds to the rotational constant for rotation around the x-axis, B corresponds to the rotational constant for rotation around the y-axis and C corresponds to the rotational constant for rotation around the z-axis. The values of A, B and C depend on the moment of inertia of the molecule and the distribution of mass around the three axes. In general, larger molecules have smaller rotation constants, since they require more energy to rotate. The rotation constants are useful in predicting the rotational spectra of a molecule, which can be measured experimentally using techniques such as microwave spectroscopy.

The B3LYP energy represents the total electronic energy of a molecule calculated using density functional theory (DFT) with the B3LYP hybrid functional. It provides an estimation of the molecule’s stability. The zero-point energy (ZPE) is the lowest possible energy a quantum mechanical system can possess. It represents the vibrational energy of the molecule at its ground state. Enthalpy is a thermodynamic property of a substance that represents the total heat content of the system. Entropy is a measure of the randomness or disorder of a system. In molecular systems, it can be influenced by factors such as molecular conformation, vibrations and rotations. Heat capacity at constant volume (Cv) is a measure of a substance’s ability to store heat energy without changing its volume; it represents the energy required to raise the temperature of the substance by one degree Celsius at constant volume. Free energy is the energy available in a system to do work. It combines the enthalpy and entropy of the system. Rot cons stands for rotation constants.

### 3.4. Antioxidant Properties

**Figure 3:**
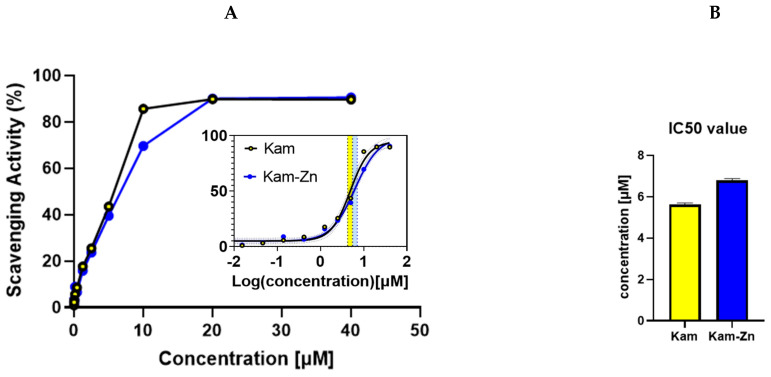
(**A**) Antioxidant activity of Kam and Kam-Zn complex in methanol solution. (**B**) IC50 value calculated from the rectilinear regression curve formula to 10 µM concentration. Yellow and blue areas represent Kam and Kam-Zn IC50 values as 95% CI (asymptotic).

The DPPH antioxidant method was employed to evaluate the IC50 values of Kam and Kam with zinc, which were found to be 5.63 ± 0.06 and 6.80 ± 0.075, respectively (Figure 3). These results indicate that Kam exhibited higher antioxidant potential than its metal–flavonoid complex. One possible explanation for the lower antioxidant potential of the metal complex with flavonoid could be due to the structural changes that occur when the flavonoid binds to the metal ion. This may affect the ability of the flavonoid to donate electrons, which is a key mechanism in the antioxidant activity of flavonoids. Additionally, the complexation with metal ions may also reduce the solubility of the flavonoids, affecting their ability to interact with free radicals in solution. Also, the binding process blocks hydroxyl groups that are essential in reactions with ROS, reducing flavonoids’ antioxidant potential. Overall, these findings suggest that the antioxidant potential of flavonoids may be altered when they form metal complexes, highlighting the importance of considering the effects of metal binding when evaluating the antioxidant activity.

### 3.5. Spectroscopy Studies and Structure Analysis

#### 3.5.1. UV-Vis Spectroscopy

**Figure 4:**
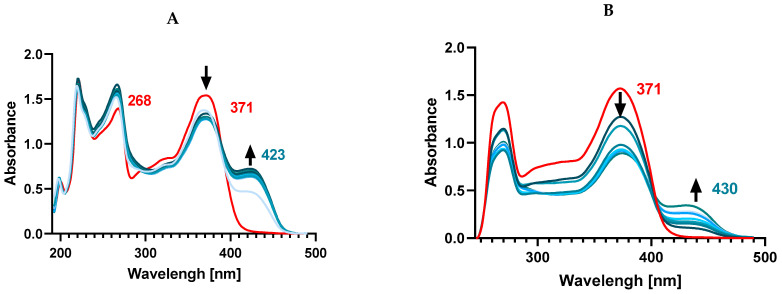
UV-Vis spectra in (**A**) methanol solution and (**B**) DMSO solution. Red lines—ligand, blue lines—metal-complex formation.

The UV-Vis spectra of both flavones and flavonols show two characteristic absorption peaks related to π → π* transitions located within ring A and ring C (around 240–290 nm, referred to as band II, benzoyl system) and within the conjugated ring B with the carbonyl of ring C (around 300–415 nm, as band I, cinnamoyl system). In a pure methanolic solution of Kam, the presence of two absorption bands was observed at 268 nm (band II) and 371 nm (band I) (Figure 4). Upon the addition of Zn^2+^ ions to the ligand solution, complex formation was observed, and a new band was formed at 423 nm. The Kam band at 268 nm undergoes a slight shift towards shorter wavelengths—266 nm, while the 371 nm band splits, resulting in a decrease in its intensity during complex formation with Zn(II).

The observed transition from 371 nm to 423 nm indicates the involvement of the cinnamoyl system in the interaction with Zn^2+^ ions and the participation of 3-OH and 4-CO groups in the formation of the complex, while the slight effect on the shift of the II band suggests the lack of involvement of hydroxyl groups located in position 5-OH of the A ring.

It was found that the addition of Zn^2+^ ions in a 1:2 ratio does not cause a further band shift compared to the spectrum obtained for the ligand and Zn^2+^ solution in a 1:1 ratio, suggesting that the ligand binds to the metal in a 1:1 ratio, which is consistent with the data presented in [36]. As they state, the 3-OH group is more acidic, and therefore, together with the carbonyl group, it will be the preferred binding site, without the involvement of the 5-OH group due to its lower acidity and steric hindrance caused by the first complexation [37]. We also conducted a similar spectral registration in DMSO to establish the binding mode of Zn^2+^ and Kam in a solvent significant for biological research. In DMSO, Kam displays its main absorption maxima at 265 nm and 372 nm, respectively. Adding Zn^2+^ ions to the ligand solution in a 1:1 and 1:2 ratio results in the disappearance of both bands present in the ligand and the formation of a band at 430 nm. This may suggest a different type of coordination compared to the methanol solution, where only the disappearance of a single band at 371 nm corresponding to the cinnamoyl system was observed. In this case, the disappearance of the 265 nm band might indicate the presence of bonds with the 5-OH group of ring A.

#### 3.5.2. Job’s Method

**Figure 5:**
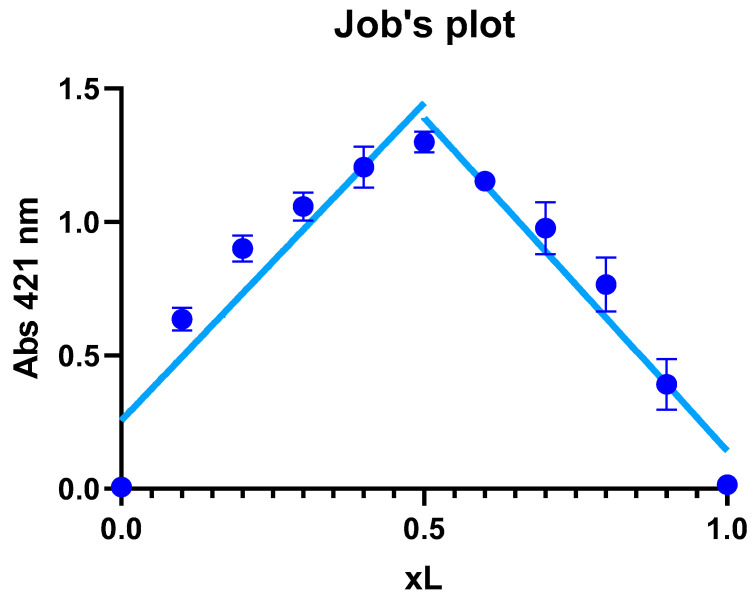
Job’s method of continuous variation in the stoichiometric ratio between Zn(II) and Kam. Mean absorbance values plotted against XL values for the investigated complex. Error bars represent standard deviations calculated from three independent measurements.

The results showed that the maximal absorption occurred at an XL ratio of 0.5 (Figure 5), suggesting that the stoichiometry of the complex formed between the zinc ion and Kam is 1:1, meaning that one Zn^2+^ ion binds with one Kam ligand to form the complex. The 1:1 ratio implies that the metal and ligand are present in equal amounts in the complex, which can be represented as structure A or B in the proposed optimized structures in the Section 3.1. These data are consistent with the results of the [11], where the structure of the Kam-Zn complex was described in a 1:1 ratio with coordinated water molecules to the metal ion.

#### 3.5.3. FT-IR

The theoretical calculations were consistent with the experimental findings, confirming the changes in the molecular vibrations of the functional groups upon complexation with zinc. The scaling factor (IR factors) of 0.98 applied to the calculated IR spectra enabled a more accurate comparison between the experimental and theoretical wavenumbers.

**Figure 6:**
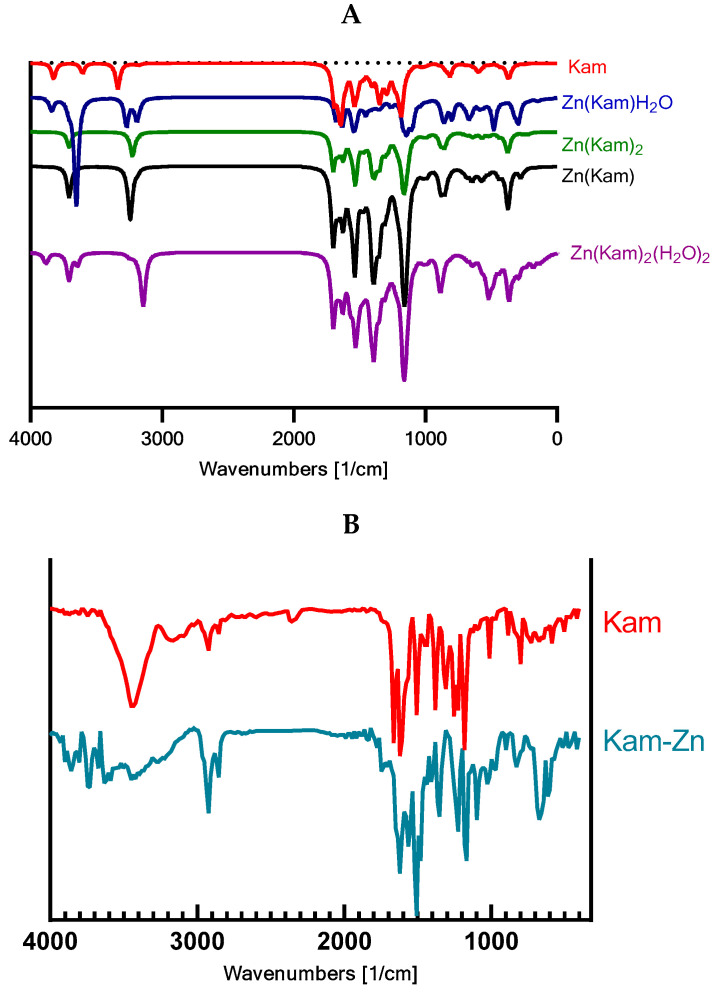
Theoretical spectra FT-IR (**A**) for Kam and proposed Kam-Zn structures. (**B**) Experimental FT-IR spectra.

The experimental FT-IR spectra of Kam and Kam–zinc complexes showed significant differences, indicating the coordination of the zinc ion to specific functional groups in the Kam molecule (Figure 6, Table 3). Based on the analysis, the zinc ion appears to be coordinated to the 3-OH and 4C=O carbonyl group. Notably, the carbonyl band observed in the Kam spectrum is absent in the Kam–zinc complex spectrum, further supporting the coordination of the zinc ion to the carbonyl group.

#### 3.5.4. Cytotoxic Properties

**Figure 7:**
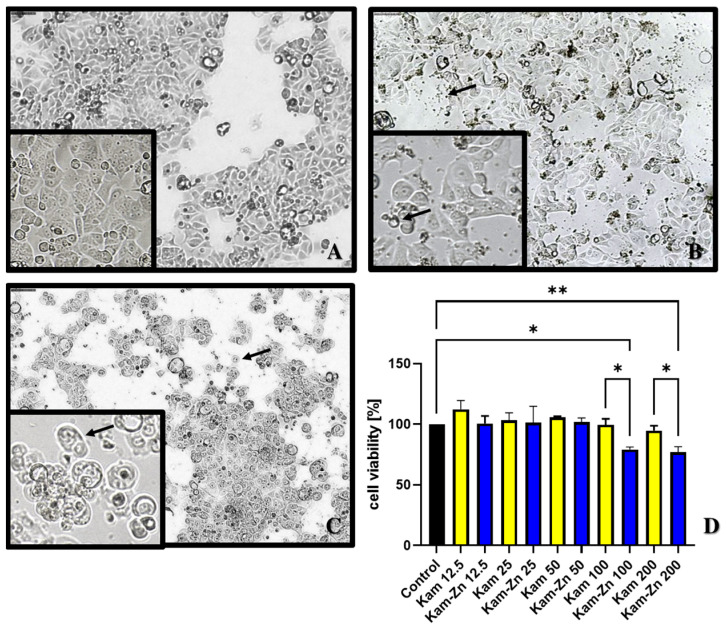
Cell morphology of the MCF-7 cell line after 24 h of incubation with the tested compounds: control (**A**), Kam 200 µM (**B**), Kam-Zn 100 µM (**C**). (**D**) Cell viability after 24 h of incubation with the tested compounds. * *p* ≤ 0.05, ** *p* ≤ 0.01.

A significant decrease in the viability of MCF-7 cell lines after 24 h of incubation was observed with the Kam-Zn complex at both 100 μM and 200 μM concentrations, which was (79.22 ± 1.96) and (76.84 ± 4.63), respectively (Figure 7D). Interestingly, comparing the cytotoxic effect of the metal complex with the ligand alone, it can be noted that at both 100 μM and 200 μM concentrations, Kam-Zn exhibits significantly stronger cytotoxic properties compared to Kam, which, in the tested concentration range, did not show significant toxicity towards MCF-7 cells.

Additionally, morphological changes were observed in the cells compared to non-treated group (Figure 7A): although Kam did not show toxicity, the cells visibly decreased in volume, and larger vesicles might suggest vacuolization in the presence of this compound (Figure 7B—arrows). In the case of Kam-Zn, a noticeable toxic effect can be observed by the cells adopting a round shape and losing adhesion to the substrate, as well as the presence of dead cells in the field of view and shrinkage (Figure 7C—arrows).

### 3.6. Antiproliferative Activity

In this study, we used non-toxic concentrations of the tested compounds, as determined by the MTT assay (12.5 μM–100 μM), to specifically observe the inhibition of cell proliferation without inducing pro-apoptotic effects.

**Figure 8:**
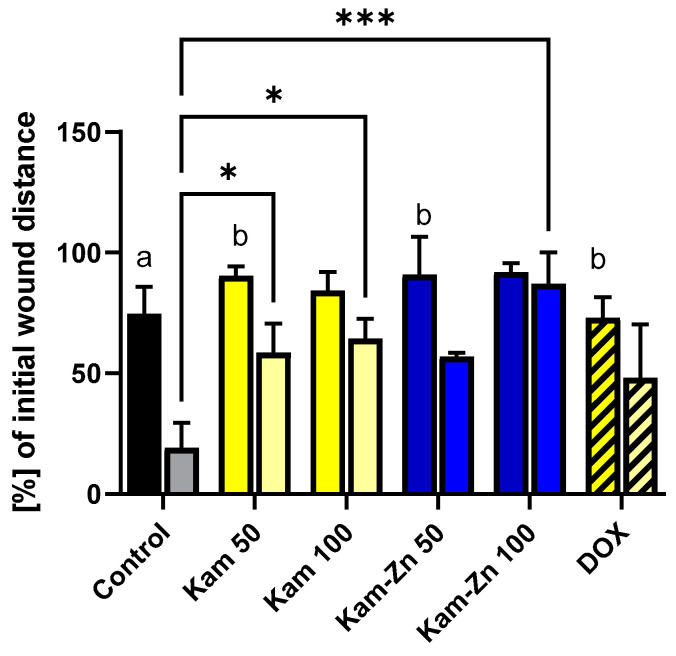
Inhibitory effects of DOX, Kam and a Kam-Zn(II) complex on the proliferation of MCF-7 cells in a wound-healing assay treated with the compounds for 16 (first bar) and 32 h (second bar). Two-way ANOVA comparison with the control group. Letter annotations denote within-sample comparisons: a (** *p* ≤ 0.01), b (*p* ≤ 0.05). Significance levels: * *p* ≤ 0.05, *** *p* ≤ 0.001.

Figure 8 demonstrates the migration and proliferation of MCF-7 cells after 16 and 32 h of incubation. In the control group, MCF-7 cells proliferated at a statistically significant rate, reducing the gap width to 19.12 ± 7.4% of the initial distance after 32 h incubation. Comparing both time points within a single tested compound, it can be observed that in Kam 100 (distance reduced by 84.86 ± 7.79 to 64.37 ± 8.24) and Kam-Zn 100 (distance reduced by 91.87 ± 3.80 to 87.04 ± 13.00), there was no statistically significant difference in the percentage change in the initial distance (16 h vs. 32 h). Interestingly, the significant difference between the distance in the control sample after 32 h and the metal complex Kam-Zn at a concentration of 100 μM (87.04 ± 13.00) indicates the effective inhibition of MCF-7 cell proliferation by this complex. Moreover, Kam-Zn treatment resulted in the inhibition of cell proliferation (no statistically significant change in distance) (Figure 8).

## 4. Discussion

This study delves into the intricate molecular dynamics and therapeutic implications of kaempferol when coordinated with zinc ions, utilizing cutting-edge computational methods and spectroscopic analyses to reveal substantial modifications in the flavonoid’s molecular structure and electronic properties. By providing a detailed characterization of how zinc alters the HOMO–LUMO gap, electronegativity and ionization potential of kaempferol, the research offers a profound understanding of how these changes potentially enhance the flavonoid’s cytotoxicity against breast cancer cells, particularly MCF-7, and moderate its antioxidant capacity. In our article, we have attempted to explain how Zn^2+^ coordination alters kaempferol’s molecular structure, specifically impacting the HOMO–LUMO gap, electronegativity and ionization potential, which are crucial for understanding the molecular basis of its altered biological activities.

The changes in electronic properties, such as HOMO–LUMO energy levels, ionization potential, electron affinity, electronegativity, hardness, softness and electrophilicity index, provide valuable insights into the reactivity of the metal complexes. The coordination of the ligand with the metal ion in structures A and B results in altered electronic properties, which can influence their reactivity and potential biological activity. In this case, the coordination of zinc to the ligand might stabilize the molecule, reducing its reactivity and antioxidant properties.

It is important to note that a higher dipole moment does not always correspond to an increase in reactivity. However, in the case of these structures, the increase in the dipole moment may lead to a decrease in reactivity due to several factors. The higher dipole moment in structure B indicates a stronger charge separation, which may lead the molecule, making it less likely to react with other molecules in its environment. An increase in the dipole moment could also affect the molecule’s ability to cross cell membranes. Generally, more polar molecules have a harder time crossing lipid bilayers, which are predominantly nonpolar. As a result, a molecule with a higher dipole moment may have reduced bioavailability, leading to a lower overall reactivity in biological systems. The energy gap (EGap) between the HOMO and LUMO orbitals may influence antioxidant properties. Smaller energy gaps often result in higher reactivity. For Kam, the EGap is 3.711 eV, while for structure A, it is 2.57 eV, and for structure B, it is 4.65 eV. The larger EGap for structure B suggests reduced reactivity, while the smaller EGap for structure A may indicate increased reactivity. However, other factors, such as dipole moment and metal coordination, might counteract this effect in structure A.

The interaction between kaempferol and zinc ions leading to altered reactivity in structures A and B could be further understood by considering how molecular dipoles impact reactivity and bioavailability. Molecules with permanent dipoles like fluoromethane interact more strongly than those relying on temporary dipoles, suggesting that kaempferol–zinc complexes with higher dipole moments may exhibit stronger interactions within biological systems [38]. Additionally, the orientation of polar bonds within a molecule can significantly influence its overall dipole moment, impacting its reactivity and interactions with other molecules [39].

Through spectroscopic studies, we observed that Zn^2+^ is coordinated by the 3-OH group and the 4-CO carbonyl group. The 5-OH group is not involved in the coordination in methanol. Zn^2+^ can form complexes with the 5-OH group in DMSO, suggesting a change in the type of coordination depending on the solvent. The cellular environment is hydrophilic, so it can be suggested that the structure present in body fluids is a complex where Zn^2+^ is coordinated by the 3-OH and 4-CO groups.

In the investigation of metal–ligand complexes for anticancer activity, the La(III) complex demonstrated superior cytotoxic effects against HepG-2 and HL-60 cell lines compared to its constituent components, attributed to enhanced π-bonding from metal–ligand chelation [17]. This complex also outperformed the chemotherapy agent 5-fluorouracil against HL-60 cells, indicating its potential as an effective anticancer agent. Additionally, the Kam-Ru complex showed significant antiproliferative effects on A549 cells, with selective cytotoxicity towards cancer cells while sparing normal fibroblast cells [40]. In bone tissue engineering, the Kam-Zn complex enhanced osteoblast activity and promoted bone formation in a zebrafish model at a concentration of 25 µM, suggesting its application in bone regeneration [41]. These results highlight the therapeutic promise of these complexes in cancer treatment and regenerative medicine, warranting further exploration in clinical settings. In our research, potential anticancer activity was observed in viability tests and cell migration assays in a breast cancer MCF-7 cell line. In the case of the metal complex, it was observed that it effectively inhibits cell migration, and the gap in the monolayer does not significantly decrease in width at either lower or higher tested concentrations, in contrast to the ligand alone—where only the higher concentration (100 µM) significantly demonstrated an inhibitory effect on proliferation. This is consistent with existing knowledge that low concentrations of kaempferol are well-tolerated and safe as a natural component of plant food [42].

Zinc complexes are also increasingly recognized for their potential in cancer therapy due to several factors. Metal-based compounds with excellent photo-physical properties show good photochemotherapeutic performance; however, the low in-depth tissue penetration of light limits their effectiveness for deeply buried tumors [43]. Encouraged by the sonosensitizing ability of traditional organic photosensitizers, researchers have explored Zn(II) complexes in photo dynamic therapy (PDT) due to their ability to assist with Lewis activation and nucleophile formation [44]. Zinc is essential for human physiology, which could mean fewer side effects compared to non-essential metal-based compounds. Additionally, zinc’s relatively non-toxic nature even at higher doses offers obvious advantages for biocompatibility, and Zn(II) complexes may target different mechanisms of action from classical platinum-based drugs. Recent studies highlight Zn(II) derivatives as potential low-toxicity anticancer agents with different cellular targets and modes of action compared to classical metal-based drugs. However, the activity of Zn coordination complexes might not be as pronounced compared to that of other metal-based derivatives due to zinc’s high cellular concentration. This suggests that the antitumor efficacy of Zn coordination complexes could be enhanced when used in combination with other chemotherapeutic agents to reduce side effects [45]. A unique feature of metal complexes in the context of anticancer therapy is their ability to activate within the tumor environment. Redox-active metal complexes can exploit the unique redox environment within cancer cells to become activated, thus inducing oxidative or reductive stress that can lead to cancer cell death. Current research confirms that this is a promising direction for the development of effective therapies, including the activation of stable forms to activate stable N-heterocyclic carbene-gold(I)-alkyne complexes, transforming them into active species that inhibit thioredoxin reductase and exhibit significant anticancer activity in treating hepatocellular carcinoma [46].

## 5. Conclusions

The coordination of kaempferol with Zn^2+^ leads to notable changes in its molecular structure, as evidenced by spectroscopy analyses and computational studies. These alterations significantly impact the compound’s electronic properties, including the HOMO–LUMO gap, electronegativity and ionization potential, suggesting a potential shift in its biological activity. Our antioxidant assays revealed that while kaempferol exhibits significant radical scavenging activity, its complexation with zinc ions slightly moderates this property. This observation underscores the complex interplay between metal ion coordination and flavonoid antioxidant capability, highlighting the need for a nuanced understanding of such interactions in therapeutic contexts. The findings of this study contribute to the growing body of literature on the therapeutic utility of metal–flavonoid complexes. By modulating the electronic and structural attributes of kaempferol, zinc ions might influence its biological activity, offering a new perspective on employing such complexes in cancer therapy, potentially enhancing efficacy or reducing side effects associated with conventional treatments.

Additionally, exploring the synergy between Kam-Zn complexes and existing cancer therapies could unveil novel combination treatments. Our current research on Kam-Zn metal complexes focuses on elucidating their biological mechanism of action, cytotoxicity and—as redox-active compounds—their impact on mitochondrial bioenergetics.

## Figures and Tables

**Table 1:** Mulliken charges of Kam and proposed structures of Kam-Zn(II) complexes calculated by Gaussian 09 using the B3LYP method.

		A	B	C	D	
Mulliken Charges	Kam	Zn(Kam)	Zn(Kam)(H_2_O)_2_	Zn(Kam)_2_	Zn(Kam)_2_(H_2_O)_2_	
Zn	-	0.558	1.110	1.102	1.082	
C2	0.651	0.124	0.100	0.113	0.097	0.104
C3	−0.245	0.138	0.152	0.168	0.155	0.139
C4	−0.145	0.224	0.191	0.244	0.214	0.245
C5	−0.510	0.221	0.180	0.215	0.209	0.210
C6	0.542	−0.556	−0.568	−0.556	−0.561	−0.561
C7	−0.260	0.413	0.396	0.412	0.411	0.412
C8	−0.633	−0.549	−0.566	−0.549	−0.552	−0.552
C9	0.968	0.286	0.251	0.280	0.277	0.279
C10	0.430	0.061	0.095	0.062	0.053	0.047
C1′	1.155	0.331	0.335	0.330	0.311	−0.341
C2′	−0.012	−0.338	−0.371	−0.339	−0.346	−0.345
C3′	−0.275	−0.334	−0.337	−0.335	−0.337	−0.336
C4′	−0.159	0.293	0.269	0.293	0.289	0.289
C5′	−0.329	−0.388	0.383	−0.391	−0.393	−0.391
C6′	−0.493	−0.341	0.384	−0.338	−0.350	−0.357
O1	0.383	−0.324	0.365	−0.322	−0.324	−0.325
O3	−0.580	−0.603	−0.715	−0.613	−0.578	−0.607
O4	−0.666	−0.586	−0.742	−0.594	−0.562	−0.559
O5	−0.536	−0.452	−0.490	−0.451	−0.460	−0.459
O7	−0.476	−0.456	−0.476	−0.456	−0.460	−0.460
O4′	−0.495	−0.458	−0.475	−0.459	−0.463	−0.467
H3	0.429	-	-	-	-	-
H5	0.445	0.421	0.407	0.424	0.419	0.422
H6	0.133	0.249	0.231	0.249	0.245	0.245
H7	0.375	0.370	0.355	0.370	0.367	0.368
H8	0.167	0.283	0.270	0.283	0.281	0.281
H2′	0.106	0.262	0.263	0.262	0.260	0.261
H3′	0.164	0.249	0.236	0.249	0.245	0.246
H4′	0.369	0.363	0.348	0.363	0.360	0.360
H5′	0.113	0.218	0.197	0.219	0.217	0.217
H6′	0.152	0.317	0.282	0.317	0.321	0.316

**Table 2:** Physicochemical descriptors obtained by theoretical chemistry method (Gaussian 09).

		Kam	A	B	C	D
			Zn(Kam)	Zn(Kam)(H_2_O)_2_	Zn(Kam)_2_	Zn(Kam)_2_(H_2_O)_2_
Method		B3LYP 6-31++G(d,p)	B3LYP LANL2DZ	B3LYP LANL2DZ	B3LYP LANL2DZ	B3LYP LANL2DZ
RB3LYP energy	[Hartree]	−1029.04	−1093.84	−1246.71	−2122.18	−2275.05
ZPE		0.23	0.21	0.26	0.43	0.48
Internal energy	Hartree	−1028.79	−1093.61	−1246.43	−2121.71	−2275
Enthalpy	Hartree	−1028.79	−1093.61	−1246.43	−2121.71	−2274.53
Free energy	Hartree	−1028.79	−1093.61	−1246.43	−2121.71	−2275
Cv	al/(mol·K)	−1028.79	−1093.61	−1246.43	−2121.71	−2274.53
Entropy	al/(mol·K)	−1028.85	−1093.67	−1246.51	−2121.82	−2274.65
Dipole moment	(Debeye)	67.33	70.58	90.37	140.72	161.82
A Rot cons	(GHz)	0.64	0.29	0.21	0.08	0.08
B Rot cons	(GHz)	0.17	0.16	0.15	0.03	0.04
C rotation constants	(GHz)	0.13	0.1	0.09	0.03	0.03
LUMO −1	eV	−0.956	−0.99	−0.66	−2.59	−2.24
LUMO	eV	−2.22	−2.6	−2.02	−2.59	−2.28
HOMO	eV	−5.93	−5.18	−2.88	−5.61	−5.16
HOMO +1	eV	−6.637	−5.64	−4.93	−5.61	−5.29
EGap	eV	3.711	2.57	4.65	3.01	2.88
Electron affinity		2.224	2.60	0.99	2.59	2.28
Ionization potential (IP)		5.935	5.18	5.64	5.61	5.16
Electronegativity	χ (eV)	4.079	3.89	3.31	4.1	3.72
Hardness	η (eV)	1.855	1.29	2.33	1.51	1.44
Softness	σ (eV)	0.269	0.39	0.22	0.33	0.35
Electrophilicity index	ω (eV)	4.485	5.88	2.36	5.58	4.81
Chemical potential	μ (eV)	−4.079	−3.89	−3.31	−4.1	−3.72

**Table 3:** Theoretical spectra FT-IR bands for Kam, proposed Kam-Zn structures and experimental FT-IR spectra.

Kam_exp_	Kam-Zn_exp_	Kam_teor_	Kam-Zn_teor_ (A)	
3434	3736	-	3630 3634	Hydroxyl (O-H) stretch
-	-	3604	-	
2914	2927	-		
2861	2856	-	-	
1661	-	1668	*-*	Carbonyl (C=O) stretch
-	-	1655	1655	
-	1626	-	1626	
1617	-	1624	-	
1565	1563	-	-	C=C stretch in aromatic rings
1505	1504	1515	1507	
-	1499	-	-	
1461	-	1461	-	
1443	-	1433	-	
-	1400	1405	1408	
-	1395	-		
1378	-	1378	1376	
-	1358	1363	1355	
1314	-	1302	1321	
1246	-	1233	-	C-O stretch
1222	1219	1208	1225	
1174	1175	1178	1178	
1093	1089	-	-	
1007	1009	1107	1103	
973	970	977	966	
888	885	893	866	
835	834	833	835	
820	-	821	-	
798	-	-	-	
-	677	-	-	
626	-	-	-	
-	666	607	-	
-	603	-	-	
592	590	594	-	
491	-	-	-	
-	443	436	-	

## Data Availability

The original contributions presented in the study are included in the article, further inquiries can be directed to the corresponding author.
